# Retroperitoneal Paraganglioma-Induced Cardiogenic Shock Rescued by Preoperative Arterial Embolization

**DOI:** 10.1155/2018/4058046

**Published:** 2018-07-05

**Authors:** N. Houari, S. Touzani, H. Salhi, M.-Y. Alaoui Lamrani, K. Ibnmajdoub, H. El Ouahabi, A. El Bouazzaoui, B. Boukatta, M. Maâroufi, K. Maazaz, N. Kanjaa

**Affiliations:** ^1^Anesthesiology & Intensive Care Unit A4, Hassan II University Hospital, Fez, Morocco; ^2^Endocrinology Department, Hassan II University Hospital, Fez, Morocco; ^3^Imaging and Interventional Radiology Department, Hassan II University Hospital, Fez, Morocco; ^4^Visceral Surgery Department, Hassan II University Hospital, Fez, Morocco

## Abstract

**Background:**

Catecholamine-induced cardiogenic shock is a rare manifestation of paragangliomas. The high mortality rate of this condition makes the immediate, multidisciplinary approach mandatory.

**Case Report:**

We report a case of an 18-year-old woman with a retroperitoneal secreting paraganglioma, complicated with a cardiogenic shock and an acute adrenergic myocarditis, requiring hemodynamic support and emergency arterial embolization prior to surgical excision, with a favorable outcome.

**Conclusion:**

Paraganglioma-induced myocarditis is rare but can be dramatic. Management requires appropriate and immediate hemodynamic support. Embolization may be an alternative to stabilize the patient prior to surgery.

## 1. Background

Paragangliomas (PG) are rare tumors originating from neural crest cells, arising at various locations along the chain of the sympathetic nervous system as well as the branchiomeric paraganglioma. Complete surgical excision remains the main available treatment and the complexity of the clinical manifestations often makes surgery part of a multidisciplinary approach. Preoperative embolization is still controversial and its role has not been established, especially in cases of paraganglioma-induced adrenergic myocarditis. We report a peculiar case that highlights the importance of preoperative embolization, to stabilize a patient with cardiac manifestations related to a catecholamine-secreting PG.

## 2. Case Presentation

An 18-year-old female patient was admitted to the endocrinology unit for assessment and preoperative management of a retroperitoneal PG. The patient's past medical history was significant for psoriasis since age 2, for which she has been getting an association of betamethasone and salicylic acid. The patient also reports a history of functional colopathy for the past 3 months. No other significant history of endocrine or tumoral conditions was reported. The patient has been suffering from recurring episodes of excessive perspiration and palpitations over the past 4 years, associated with other symptoms of hypertension such as headaches and tinnitus. The patient also reported multiple episodes of recurrent right-sided abdominal pain worsening over the past year.

**Figure 1:**
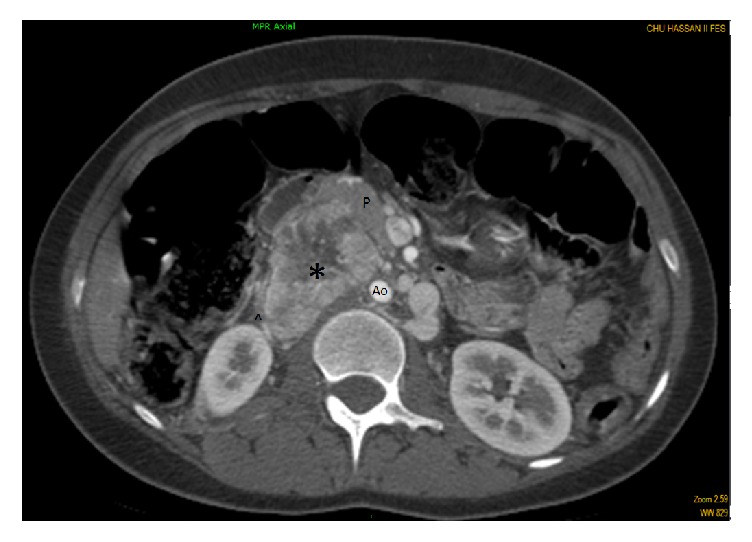
A contrast abdominal CT scan showing a 7.0 x 5.0 cm heterogeneous mass (black asterisk) located between the abdominal aorta (Ao) and the inferior vena cava, pushing the head of the pancreas (p).

Her physical exam upon admission was normal with a BMI (Body Mass Index) = 21.8 kg/m^2^, a BP (blood pressure) = 130/90 mmHg bilaterally (no postural hypotension was noted), and a heart rate (HR) =88 beats per minute (bpm). The cutaneous examination showed facial erythrosis and eczematous lesions of the upper and lower extremities. No pigmentation disorders nor cutaneous superficial neurofibromas were noted. EKG analysis showed a sinus rhythm and a left ventricular hypertrophy (LVH). An abdominal computed tomography (CT) scan revealed no adrenal abnormalities but a 7.0 x 5.0 cm tissular-like retroperitoneal mass in contact with the abdominal aorta and the inferior vena cava, intimately related and displacing anteriorly the head of the pancreas (**Figure 1**). Abdominal MRI and elevated urinary methylated metabolites of catecholamines (Metanephrine = 3.2 *μ*mol / 24h (normal range (NR) 0.2 to 1), Normetanephrine = 47.5 *μ*mol / 24 h (NR: 0.4 to 2.1)) confirmed the diagnosis of catecholamine-secreting retroperitoneal PG.

Twenty-four hours after admission, the patient developed a cardiogenic shock. Her initial vital signs were as follows: Glasgow Coma Scale (GCS) = 14 (E4 V4 M6), BP = 82/46 mmHg, HR = 150 bpm, a respiratory rate at 25 cycles per minute with a SpO2 (peripheral oxygen saturation) at 60% with cold distal extremities and cyanosis. Capillary blood glucose was 150 mg/dl. Fluid resuscitation using 500 ml of Ringer Lactate solution and oxygen through high concentration mask were both promptly started and the patient was transferred to the ICU.

On admission, clinical assessment showed signs of heart failure, elevated troponin, and lactates markers and an EKG revealed sinus tachycardia with a frequency of 130 bpm. The patient was hypoxic with a SpO2 at 95% under 9 L/min of oxygen. Two right femoral central lines were placed (venous and arterial) and the patient was started on Noradrenalin (at a rate of 0.5 *μ*g/kg/min). An echocardiography revealed a global hypokinesia with a left ventricular ejection fraction (LVEF) at 15%, requiring the patient to be put on 15 *μ*g/kg/min of Dobutamine as inotropic support.

**Figure 2:**
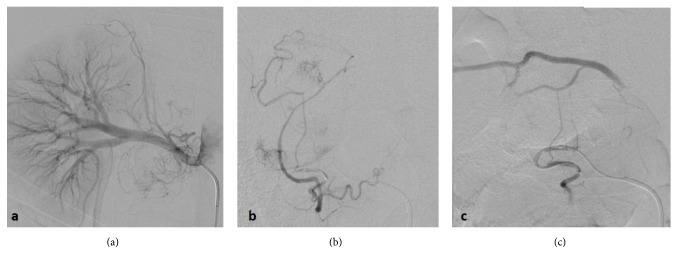
Angiography showing several feeding arteries arising from the right renal artery (a) and the 3rd right lumbar artery (L3) (b). Successful embolization of several arterial arteries: e.g., the 3rd right lumbar artery (c).

Seeing how the patient was in no shape to undergo open surgery, we opted for a preoperative radiological percutaneous transarterial embolization (TAE), under local anesthesia. It consisted of coiling of four major feeding vessels, 2 arising from the right renal artery and 2 more from the lumbar arteries (L2 and L3), using hydrogel with Polyzene®-F coating microparticules (**Figure 2**). The patient was gradually weaned off catecholamines during embolization. She was completely weaned off vasoactive drugs in the next 24 hours. 48 hours after embolization, a follow-up echocardiography showed an overall normokinetic left ventricle (LV), an improved FEVG at 50%, and an aortic VTI (velocity time integral) at 20m/s. Lactate levels went back to normal over the next 48 hours. In the postembolization period, the patient experienced several nonsymptomatic hypertension peaks (systolic blood pressure (SBP) = 220mmHg) and received intravenous nicardipine at a rate of 5 mg/H. The extremities warmed up and the daily urine output was maintained.

**Figure 3:**
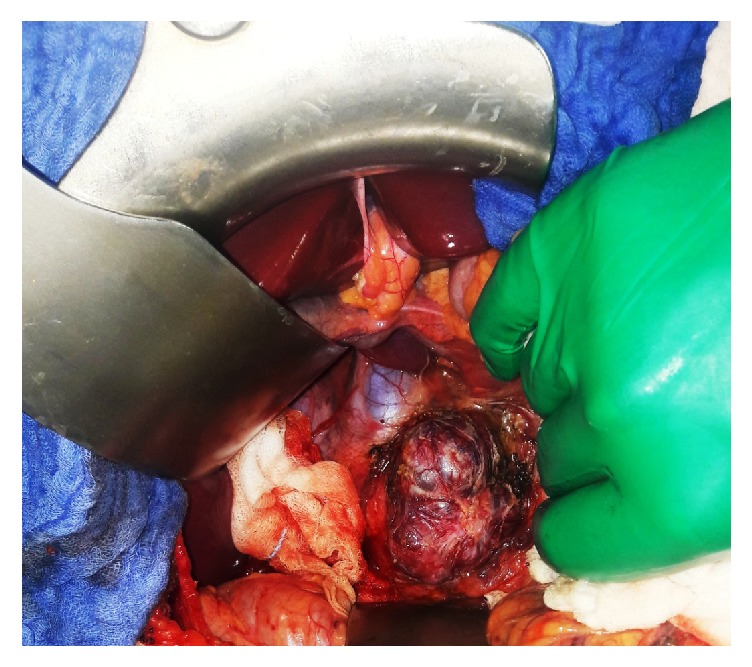
Surgical site showing the presence of the paraganglioma with intimate contact with the abdominal aorta and the inferior vena cava after mobilization of the duodenum.

**Figure 4:**
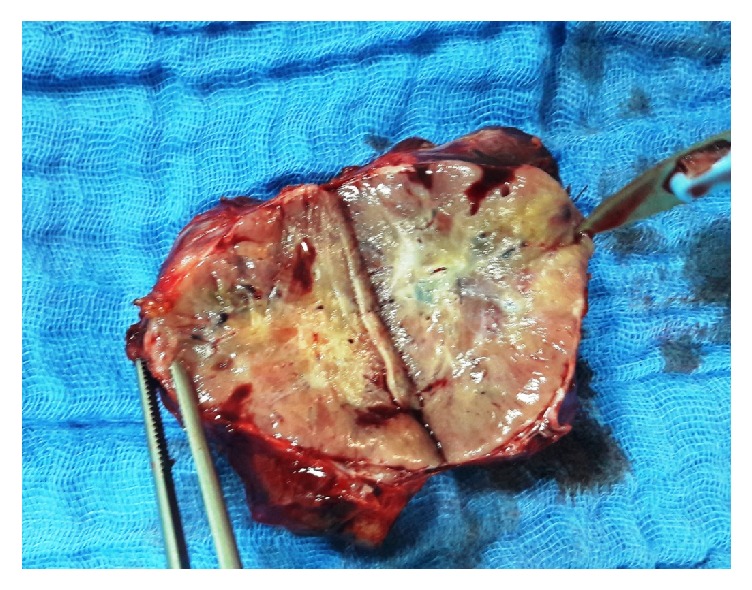
Cut gross specimen showing a tan colored mass.

Three days after embolization, the patient was taken to the operating room for the tumor to be surgically removed through a right subcostal approach. Perioperative exploration found a retroperitoneal soft encapsulated tumor measuring 7.0 cm in diameter, in intimate contact with the abdominal aorta, the right renal vein, and the inferior vena cava (**Figure 3**). Embolization caused no complications. The tumor's adhesions were carefully released from adjacent organs including the right kidney, the duodenum, and the head of the pancreas. There was no need to place a double J ureteral stent preoperatively. Upon manipulation of the mass, there was moderate elevation in the blood pressure that was controlled with intravenous propranolol and nicardipine. A complete resection of the tumor was performed and no adjacent organs were removed or injured during the surgery (**Figure 4**).

The patient experienced episodes of hypotension after the tumor excision and received intravenous noradrenaline at a rate of 0.3 *μ*g/kg/min. The patient was then transferred back to the ICU and extubated in fast track manner. She was weaned off vasoactive drugs in the following 6 hours.

**Table 1** sums up the lab work-up of the patient during her stay.

During close ICU monitoring, the patient presented several episodes of hypoglycemia and hypokalemia and was given intravenous supplementation of glucose and potassium. A postoperative echocardiography showed a hyperkinetic myocardium with an enlarged LV, an IVS (interventricular septum) measuring 13 mm, a lateral wall measuring 14 mm, and a much improved LVEF at 70%.

The patient was subsequently discharged after spending 4 days in the ICU and transferred to a general surgery service. Pathological report of the tumor mass confirmed the diagnosis of benign extra-adrenal PG. A one-month echocardiography follow-up noted nothing significant. She now completely recovered and is back to her daily life.

## 3. Discussion

Paragangliomas (PG) are rare tumors derived from neural crest cells, whose origins may vary along the chain of the autonomic nervous system [1]. Most of PG are located in the head and the neck while deriving from the parasympathetic nervous system and rarely produce catecholamines. On the contrary, most of the PG deriving from the sympathic system are located in the abdomen, produce catecholamines, and are unique, sporadic, benign, and more common in middle-aged women [2].

Patients with functional PG, such as pheochromocytoma, usually present a triad of clinical symptoms (Menard's triad): cephalgia, tachycardia, and diaphoresis, these symptoms being mainly related to high levels of catecholamines in blood. Putting a label on this pathology can sometimes be challenging, due to the variability of the clinical presentation, including a variety of life-threatening cardiovascular syndromes, such as hypertensive crisis, acute myocardial infarction (AMI), acute heart failure, shock or profound hypotension, arrhythmia, myocarditis, or cardiomyopathy [3, 4]. The cardiovascular complications seem mainly related to the massive dumping of high levels of catecholamines in blood, especially after the tumor's blood supply has been cut off [5]. Increased catecholamine levels may cause structural and biochemical damage to the myocardium, leading to catecholamine-induced cardiomyopathy [6]. This variety of clinical features can explain why the differential diagnosis is often demanding.

There are reports of many cases, as for the case presented herein, of catecholamine-induced shock. Several factors could explain its intricate mechanisms, the main one being the abrupt interruption of catecholamine secretion by the neuroendocrine tumor after a long period of catecholamine-induced hypertension in a patient with a limited circulatory volume and desensitized adrenoceptors [7]. Other factors include the direct toxicity of catecholamines derivatives [8] and the negative inotropic effect of hypocalcemia (as the calcium is sequestrated in the myocytes' sarcoplasm) [9].

AMI cases have also been linked to these tumors. Less than half the patients having had catecholamine-induced myocardial infractions, especially those with preexisting cardiovascular conditions, showed abnormal coronarography [4], which could suggest that myocardial injury in patients with previously healthy hearts is either due to a hemodynamic compromise (an increase in myocardial oxygen demand with a decrease in myocardial oxygen supply) or due to the immediate deleterious effects on cardiac function of catecholamines (accelerated apoptosis and fibrosis) [7]. There were no indications to perform a coronarography in our case given the patient's young age, the absence of preexisting cardiovascular conditions, and the evident, widespread myocardial lesions in the echocardiography findings.

High catecholamine levels lead to faster lipid mobilization, calcium overload, free radical production, and an increased sarcolemmal permeability [10], thus causing either myocarditis through direct myocardial damage or hypertrophic/dilated cardiomyopathy. The latter, far more frequent, can occur at any age, even during childhood [6]. In most cases, myocardial damage caused by catecholamine-induced cardiomyopathy is reversible through proper treatment and/or surgical excision. Treatment consists of continuous inotropic support, and in some cases (during the acute phase of the cardiogenic shock) even the use of circulatory assistance such as Extracorporeal Membrane Oxygenation (ECMO) and Intra-Aortic Balloon Pump (IABP) [9, 11, 12]. Some patients improved right after medication onset, and others required up to several months for their symptoms to resolve [7].

Preoperative arterial embolization of pheochromocytomas or paragangliomas is not routinely performed because of the theoretical risk of catecholamine release in circulation resulting in hypertensive crisis. However, it can be performed under strict monitoring to reduce vascular supply in large, hypervascularized tumors, thus facilitating surgical excision [13–16]. However, there are no indications that this procedure significantly reduces perioperative blood loss, surgical time, or hospital stay [17, 18].

To the best of our knowledge, arterial embolization has only been described once in cases of catecholamine-induced refractory cardiogenic shock. It aimed to wean a patient in cardiogenic shock from emergency extracorporeal life support (ECLS) as a bridge to final surgery [11]. After the procedure, the patient improved, and ECLS was weaned in 48h without complications, and adrenalectomy was performed after 10 weeks.

Considering the severity of the clinical manifestations and the fact that the patient's condition was already labeled, we decided to carry out an emergency arterial embolization under strict monitoring right after the first signs of cardiogenic shock. This procedure allowed our team to wean the patient off inotropic and vasoactive drugs in a matter of hours.

There is no consensus for the exact delay of surgery after an episode of catecholamine-induced cardiomyopathy. It can range from a few days to several weeks [9, 11]. Some authors report carrying out surgery during the acute phase, with no significant perioperative complications and an improved postoperative cardiac function [12, 19]. In our case, surgery was scheduled right after stabilization to avoid a full-blown relapse.

## 4. Conclusion

Our case is a good example of how challenging the diagnosis of PG can be, especially when it lacks the typical clinical presentation, and is manifested by cardiovascular events, as it is the case with pheochromocytoma/paraganglioma-induced adrenergic myocarditis. Management requires appropriate and immediate hemodynamic support and circulatory assistance may prove mandatory when it comes to refractory cardiogenic shock. Surgical resection after adequate medical preparation is still the main treatment and it is necessary for histological assessment, but preoperative endovascular embolization can prove to be a great, less risky, alternative to stabilize patients prior to surgery.

## Figures and Tables

**Table 1:** The patient's lab work-up evolution during the hospital stay.

	**Admission Day -1 (Endocrinology Unit)**	**Day 0 (Cardiogenic Shock)**	**H+6 (After Embolization)**	**Day 2**	**Day 4 (Tumor Resection)**	**Postop Day 1**
**Hemoglobin** (G/DL)	16	16.9	12	10.4	11	10.2
**Leukocytes **(E/MM^3^)	8750	22210	9950	9220	8980	8020
**Platelets** (G/L)	687	512	373	316	270	294
**PT**	70	64	81	97	-	95
**PTT**	31.5	30/30	30	31	-	38.5
**Glucose** (G/L)	1.33		1.85			
**Albumin**	49	41	38	32	31	29
**Protein**	82	67	63	60	52	53
**Urea**	0.35	0.59	0.25	0.16	0.08	0.07
**Creatinine** (MG/L)	7	9	7	5	5	5
**CRP** (MG/L)	1	3	18	45	78	125
**Sodium** (MMOL/L)	140	137	132	131	134	133
**Potassium** (MMOL/L)	4.4	5.5	4.6	3.3	2.9	4.5
**Chloride**	101	97	99	102	101	105
**Calcium** (MG/L)	106	89	96	-	79	85
**Magnesium**		24	21			
**Phosphorus**	43					16
**Alkaline Reserve**	26	20	25	21	21	21
**AST**	63	174	180	78	53	49
**ALT**	54	362	502	351	177	138
**CK**		204				
**CK-MB**		201				
**Troponin**	-	1.96	1.1	0.73	0.46	0.18
**PH**		7.5				
**PACO2 **		30.2				
**HCO3-**		23.6				
**PAO2**		150				
**Arterial Lactate**		1.25	0.43			

## References

[1] Tischler A. S. (2008). Pheochromocytoma and extra-adrenal paraganglioma: Updates. *Archives of Pathology & Laboratory Medicine*.

[2] Erickson D., Kudva Y. C., Ebersold M. J. (2001). Benign paragangliomas: clinical presentation and treatment outcomes in 236 patients. *The Journal of Clinical Endocrinology & Metabolism*.

[3] Kanjaa N., Khatouf M., Elhijri A. (1999). Phéochromocytomes. Formes graves et inhabituelles. *Annales Françaises d’Anesthésie et de Réanimation*.

[4] Bouazzaoui A. E., Hammas N., Houari N. (2015). Syndrome coronaire aigu: un mode de révélation peu fréquent du phéochromocytome. *Pan African Medical Journal*.

[5] Charon P., Hamwi A, Laigneau P., Beroud P. (1991). Forme pseudo-coronarienne de rupture hémorragique d’un pheochromocytome. A propos d’une observation. *Annales De Cardiologie Et D'Angeiologie*.

[6] Kizer J. R., Koniaris L. S., Edelman J. D., Sutton M. G. S. J. (2000). Pheochromocytoma crisis, cardiomyopathy, and hemodynamic collapse. *CHEST*.

[7] Prejbisz A., Lenders J. W., Eisenhofer G., Januszewicz A. (2011). Cardiovascular manifestations of phaeochromocytoma. *Journal of Hypertension*.

[8] Ganguly P. K., Beamish R. E., Dhalla N. S. (1989). Catecholamine cardiotoxicity in pheochromocytoma. *American Heart Journal*.

[9] Sojod G., Diana M., Wall J., D'Agostino J., Mutter D., Marescaux J. (2012). Successful extracorporeal membrane oxygenation treatment for pheochromocytoma-induced acute cardiac failure.. *The American Journal of Emergency Medicine*.

[10] Adameova A., Abdellatif Y., Dhalla N. S. (2009). Role of the excessive amounts of circulating catecholamines and glucocorticoids in stress-induced heart disease. *Canadian Journal of Physiology and Pharmacology*.

[11] Vagner H., Hey T. M., Elle B., Jensen M. K. (2015). Embolisation of pheochromocytoma to stabilise and wean a patient in cardiogenic shock from emergency extracorporeal life support. *BMJ Case Reports*.

[12] Ritter S., Guertler T., Meier C. A., Genoni M. (2011). Cardiogenic shock due to pheochromocytoma rescued by extracorporeal membrane oxygenation. *Interactive CardioVascular and Thoracic Surgery*.

[13] Rosing J. H., Brooke Jeffrey R., Longacre T. A., Greco R. S. (2009). Massive extra-adrenal retroperitoneal paraganglioma: Pre-operative embolization and resection. *Digestive Diseases and Sciences*.

[14] Morita S., Furuta Y., Honma A., Suzuki F., Fujita K., Fukuda S. (2008). Preoperative Embolization and Postoperative Complications of Carotid Body Tumors. *Nippon Jibiinkoka Gakkai Kaiho*.

[15] Nakamura F., Silva R. A., Santos V. P., Razuk Filho Á., Caffaro R. A. (2010). Embolização pré operatória no tratamento de paraganglioma abdominal: relato de caso. *Revista do Colégio Brasileiro de Cirurgiões*.

[16] Shakir M., Blossom G., Lippert J. (2012). Anterior mediastinal paraganglioma: A case for preoperative embolization. *World Journal of Surgical Oncology*.

[17] Boedeker C. C., Ridder G. J., Schipper J. (2005). Paragangliomas of the head and neck: Diagnosis and treatment. *Familial Cancer*.

[18] Miranda E. D. P., Lopes R. I., Padovani G. P. (2016). Malignant paraganglioma in children treated with embolization prior to surgical excision. *World Journal of Surgical Oncology*.

[19] Vandwalle J., Spie R., Jarry G., Agaesse V., Petit J., Saint F. (2010). Phéochromocytome et défaillance cardiaque : une indication exceptionnelle de surrénalectomie en urgence ou semi-urgence. *Progrès en Urologie*.

